# Bridging the Gap: Parent and Child Perspectives of Living With Cerebral Visual Impairments

**DOI:** 10.3389/fnhum.2021.689683

**Published:** 2021-07-08

**Authors:** Trudy Goodenough, Anna Pease, Cathy Williams

**Affiliations:** ^1^Centre for Academic Child Health, Bristol Medical School, University of Bristol, Bristol, United Kingdom; ^2^Bristol Eye Hospital, University Hospitals Bristol and Weston NHS Foundation Trust, Bristol, United Kingdom

**Keywords:** cerebral visual impairment, children, qualitative, vision, visual impairment

## Abstract

Cerebral Visual Impairment (CVI) is an umbrella term which includes abnormalities in visual acuity, or contrast sensitivity or colour; ocular motility; visual field and the conscious and unconscious filtering or processing of visual input. Children with CVI have specific needs and problems relating to their development from infancy to adulthood which can impact on their wellbeing. Recent research indicates the complexities of living with CVI but there remains limited information of the full impact of CVI on families’ everyday lives. The qualitative interviews reported here explored families’ experiences to discover the impact of CVI on all aspects of everyday life. Parents and children (aged 6–18) were invited to participate in semi-structured interviews, either face to face, by phone or video call between January 2018 and February 2019. Topics covered everyday practicalities of living with CVI, focusing on challenges and what worked well at school and home. Interviews were audio-recorded and subject to thematic analysis to look for patterns across the data. Twenty families took part in interviews, with eight children/young people within those families contributing interviews of their own. Four themes were developed from the interviews: (1) Assessment and understanding implications of CVI, (2) Education, (3) Family life, (4) Psychological wellbeing and quality of life. The interviews provide valuable insights into the impact of living with CVI and highlight the need for more awareness of the condition among professionals in both health and education settings.

## Introduction

Damage or poor function in areas of the brain related to vision can lead to cerebral visual impairment (CVI) (Dutton, 2003; Fazzi et al., 2007; Philip and Dutton, 2014). Cerebral visual impairment may include loss of peripheral or central visual fields; loss of control over eye movements; difficulty seeing at low contrast and difficulties with visual recognition of objects, shapes or people.

Cerebral visual impairment in children manifests in various ways; including being unable to find things in a cluttered scene; bumping into things; inability to copy from the class whiteboard to their own workbooks or difficulty controlling their eye position effectively to keep focussed on a task (Philip and Dutton, 2014). In primary school aged children, when not fully recognised and understood, these difficulties may be interpreted as lack of understanding, clumsiness or inattention, or as social and communication difficulties particularly if the child has developmental problems (Swift et al., 2008).

CVI has been reported as the main cause of childhood vision impairment in high income countries and as on the rise in low-income countries (Lueck et al., 2019; Teoh et al., 2021). Our recently published study on the prevalence of CVI in primary school age children suggested that CVI may affect as many as 3.4% of children, with the majority of these already identified as needing extra help at school (Williams et al., 2021). Children with developmental problems and disabilities, who tend to have higher rates of CVI, also have increased risks of additional mental health problems such as anxiety and depression (Davies et al., 2013).

Recent work with families where children and young people have visual impairments demonstrates the importance of listening to and understanding families’, children’s, and young people’s experiences (Tadić et al., 2015; Liebermann et al., 2017; Anderson et al., 2019). Two surveys (initial and follow up) of parents of children with CVI, demonstrated persistent difficulties with obtaining a diagnosis and access to appropriate support (Jackel et al., 2010; Jackel, 2019). The aim of the current study was to understand the broad impacts of CVI for families using qualitative interviews. Our findings contributed to the development of an intervention in English primary schools to support children with CVI, and a set of core outcomes for future researchers to consider when designing interventional studies.

This study was part of the CVI Project^1^, a 5-year programme of work involving interlinked studies which aim to increase understanding about CVI and how best to help affected children.

## Materials and Methods

Parents of children and young people with a diagnosis of CVI, given by a professional, were eligible to participate. Children/young people (CYP) aged 6–18 years were also invited provided their parent/carer or a trusted adult was present for the interview.

Diagnoses of CVI were inclusive, we did not assess children or young people for CVI but accepted parental understanding and report that their child had been given this diagnosis by a professional.

To ensure a range of ages and physical capabilities, purposive sampling identified younger children (6–11 years old) and older (12–18 years old) young people, and those with or without a diagnosis of cerebral palsy.

Families were recruited via: Specialist Teachers for Vision Impairment (QTVIs); The WESC Foundation Specialist Centre for Visual Impairment, Exeter, and the CVI Society – a UK parent support group for families of children with CVI^2^. Recruitment was supported by inviting initial contacts to snowball information to other interested groups and individuals.

Interviews within qualitative research provide the opportunity for in-depth exploration of participants’ experiences of their world. Semi-structured interviews while guided by interview topic guides allow for flexibility, offering the participants time and space to discuss and develop the areas that are important to them within the interview structure (Ritchie and Lewis, 2003; Green and Thorogood, 2018). Topic guides and interview methods for this study were developed collaboratively with the CVI Project parent and professional research advisory groups and the Family Faculty at Peninsula Childhood Disability Research Unit at University of Exeter Medical School^3^. Both groups of advisors represented a wide range of experience, including ophthalmologists, orthoptists, special educational needs teachers; community paediatrician and educational psychologist, researchers, and a range of families who had children with CVI and other neurodisabilities.

Families contributed ideas for which areas of daily life to investigate, including asking families to talk about their experiences of living with CVI including what their children enjoy and what they avoid as a way of discovering possible difficulties and how these are addressed and in practical terms offering parents and their children maximum flexibility in timing and mode of interview.

Parents’ interviews focussed on day-to-day life for families living with CVI, covering school, home, family and health and wellbeing; practicalities of living with CVI; past support received and what would help in the future.

Children’s and young people’s interviews covered what they enjoyed about school, home, family life and hobbies; support at home and school including what they liked about this support. Where appropriate, creative activities were offered to help elicit the views of children, including drawing, colouring, and tablet-based art activities. Care was taken to be sensitive to the children’s and young people’s needs during the interview including being aware when their behaviour might indicate that they wanted to pause or stop.

Following the first interview, the research team reviewed both the interview transcript data and the practicalities of the interview process, to confirm that the topics and approach were flexible and gave families the best opportunity to share their experiences.

Interviews were conducted according to participants’ preference either face to face in a location chosen by them; by telephone, or video call, and the audio was recorded with participant’s verbal and written consent. For children and young people’s interviews, consent was obtained from parents, and children and young people agreed that they would like to take part.

Interview audio recordings were transcribed verbatim, anonymised, checked for accuracy, and imported into NVivo V.11. Braun and Clarke’s 6 stage approach to thematic analysis was applied to the transcripts (Braun and Clarke, 2006). This included: initial familiarisation with the data allowed for submersion in the topic area; systematic coding across the transcripts to identify data relevant to each node; nodes were then collated into relevant themes and then the nodes within each theme were revisited to check the goodness of fit within each theme and coded extract. Initial analysis was carried out by two researchers who studied the interview data alongside the interview topic guide to identify the nodes and themes within the interviews. The first two transcripts were independently coded by two researchers to cross check the coding and early development of themes. Themes were discussed until a consensus on the description of key themes and subthemes was reached.

## Results

Twenty families took part in the interviews. Within these families, eight children and young people contributed interviews of their own. As multiple approaches to recruitment were used, it is not possible to report how many people were exposed to the study invitation or calculate a response rate. The majority of participating families lived in England (16), with fewer from Scotland (1), Northern Ireland (2), and Eire (1). Table 1 summarises the ages of the children/young people described in the parent interviews, and whether parents described their child as having cerebral palsy or registered as partially sighted/blind.

**TABLE 1:** Ages of the children described in the parent interviews and reported diagnosis of cerebral palsy or registered as partially sighted/blind.

Ages of children who were the focus of parent interviews	Number of children in each age group	Child’s Gender		Cerebral palsy diagnosis reported by parent	Registered Blind or partially sighted reported by parent	Number of children interviewed in each age group
		
		M	F			
3–5 years	2	1	1	0	1	0
6–11 years*	13	8	5	6	2	4
12–17 years*	7	4	3	2	1	4
Total	22	13	9	8	4	8

*In 2 families there was more than one child with CVI.

Twenty parent interviews were completed between January 2018 and February 2019. Seven parent interviews were by telephone, 13 face to face. One parent was interviewed both by phone and contributed again within her child’s face to face interview. Of the 8 children/young people interviewed, 1 was by video call, 1 telephone and 6 were face to face. Parent interviews lasted 45 min -2 h, child/young people’s interviews were expectedly shorter, lasting a maximum of 30 min. Additional diagnoses for the children, as reported by parents, included: premature birth; cerebral palsy; chromosomal abnormalities; epilepsy; foetal alcohol syndrome; global developmental delay; and autism. Four children/young people were registered as partially sighted or blind.

### Themes

Discussions flowed across the interview topic areas, interweaving experiences from home and school presenting a comprehensive picture of the impact of CVI on families’ everyday lives. A brief topic guide can be found in Supplementary Material A.

The four key themes and associated subthemes generated are summarised in Table 2, and described below with illustrative quotes. Children’s quotes are included wherever possible, more often representing older children’s/young people’s experiences as they were better able to reflect on how their visual challenges affected everyday life. Conversations with younger children provided insights into what they enjoyed or found difficult in everyday life, with their contributions often leading to further discussion and interpretation by their parent. A table with additional illustrative quotes for each theme can be found in Supplementary Material B.

**TABLE 2:** Themes and subthemes.

Themes	Subthemes
Assessment and understanding implications of CVI	
	Recognition of CVI as a diagnosable condition
	Professional knowledge
	Assessment
Education	Knowledge
	Assessment
	Additional diagnoses
	Learning and adaptations
	Training
	Specialist support
Family life	
	Information
	Support
	Life at home and the outside world
	Key people “enablers”
	Supporting independence
Psychological wellbeing and quality of life	
	Reduction of anxiety
	Meltdowns
	Increased social inclusion
	Reduction in frustration
	Self-esteem

**Key themes**

1. Assessment and understanding implications of CVI
2. Education
3. Family life
4. Psychological wellbeing and quality of life.

#### Assessment and Understanding of Implications of CVI

##### Recognition of CVI as a diagnosable condition

Parents reported that CVI was not always recognised as a “real” condition, without this recognition parents described challenges accessing the support that their children needed.

“*But a lot of paediatricians don’t believe that CVI is diagnosed, it’s a non-diagnosis for them, so that’s an issue in itself.*” *Parent 012*

Parents also described how receiving a diagnosis of CVI could transform the environment for their child.

“*So the only thing that let us down was the ophthalmology department*…*I had to explain every single time that [child]couldn’t communicate very well, almost just feel like you’re being fobbed off. One woman actually shouted at [child] because she wasn’t doing what she was told*…

*Researcher: What was it she couldn’t do?*

*Just the tests they were asking her to do, her attention was that of a gnat, so me and my husband sat down trawling through papers and papers on Google, and came across thankfully [name of professional] and then she’s been our saviour, took [child] to [clinic], literally first meeting [professional] asked all the questions, she asked me the questions first, [child] walked in she was like she’s got CVI, and that was it: [child] sleeps in the top bunk, because we put a tunnel over it, so when she’s in her bed there’s nothing over and that made a massive difference. We made a sensory room under the stairs*…*a little space to go where she had music, and she put lights on. All toys more tactile so that she could actually get out and play with them, she didn’t want to play with anything, because everything for kids is visually bright and lovely, take that way, give it a bit more tactile and not so bright and yeah she plays.” Parent 017*

##### Professional knowledge

Parents wanted all professionals assessing their child to fully understand possible impacts of CVI so parents could access the information and support that they and their child required.

*“very few people, doctors, professionals, look at his vision impairment from a neurological perspective I feel, it’s all about can he see this, well yes he can actually see it, he knows it’s there, but whether his brain can convert that into any meaningful message or whatever it needs to be I don’t think the professionals that we are in touch with understand CVI enough for that”. Parent 010*

*“It’s been really nice, [Sense^∗^ worker name] do a home visit, and just having that chance to talk to somebody who understands vision and the visual problems, because you don’t get that from a hospital appointment about their eyes, because you’re just testing them aren’t you? But it is really important really to have*… *those visits from [Sense worker] and the Guide Dogs^∗∗^ I can draw on them and that, but[if] I’d never had them that would be quite a big gap I think, and the Great Ormond Street neuro and psychological assessment*… *this guy had never met my kids before, he had them for an hour, he totally sussed them out in that hour. He seemed to have such a good grasp of what their difficulties were and what their strengths were. So having some sort of assessment like that is important because you want to understand what’s going wrong, and I think CVI is quite difficult to unravel and understand. It’s not like with vision you’ve got*… *with long and short sighted you’ve got numbers haven’t you to tell you this is exactly how short sighted they are, and if they’re hearing impaired you know exactly which hertz they can hear, but with CVI it’s not that straightforward.”. Parent 027^∗^*Sense: a UK charity which supports people living with complex difficulties. https://www.sense.org.uk/about/.

^∗∗^Guide Dogs: a UK charity that provides support for people with sight loss. https://www.guidedogs.org.uk/about-us/.

One young person suggested her condition should be redefined so that professionals would understand the challenges she faced:

*“If I was a doctor, I would change it to CCI, cerebral cognitive impairment*…*.There’s something called LD, a learning disability, and there’s no such thing as CCI though, because it’s not just about what you see stuff, it’s about how you think, how you process stuff, how you understanding what people are saying to you.*” *CYP 006*

##### Assessment

Early screening or assessment was described as vital to identify and manage CVI immediately any visual processing issues were suspected. Parents also emphasised the importance of incorporating *“*functional vision” testing within comprehensive assessments where their child’s behaviour or additional diagnoses might suggest CVI was present.

*“I think that’s the key thing because there’s so much confusion between autism and CVI specifically because of the poor eye contact, and it seems to be as soon as there’s anything wrong with the child they get labelled autistic because that’s all people know. So I think the early screening is really important because if he had been misdiagnosed all the wrong strategies would have been put in place.*” *Parent 028*

In addition, parents noted the difficulties that arose when individual assessments were completed in isolation, highlighting the need for improved communication between all professionals involved with the child and family.

*We need to have people working together. That’s the biggest problem she has had all her life is that none of these professionals sit down in a room to discuss*…*.we’ve had physio, okay this is to do with walking, we’ve had a physio recommend she carries bags on her sticks, now primarily we’re talking someone with balance issues, that’s like me giving you shopping bags to ride a bicycle with. We need to get people sitting in a room all talking to each other, because one aspect impacts another, impacts another*…*.We have EH [Education Health Care Plans] stating she needs occupational therapy, stating she needs physio, and you’ve got them all working singularly. It’s ridiculous, why is that so hard? Parent 006*.

#### Education

Discussions about education (at home and within school/college settings: specialist and mainstream), demonstrated the multifaceted nature of the families’ experiences. These experiences highlighted the importance of staff understanding CVI; the education assessment process; parents’ involvement in their child’s education and child’s/young person’s experiences of their educational settings.

##### Knowledge

Parents wanted schools to understand CVI and how it affected their child’s learning and school life. Particularly how additional diagnoses might compound CVI; benefits of individualised adaptations to support learning and the importance of training for all staff and professionals working within schools.

*“I would like to see the VI service incorporate CVI, I think it’s crucial, because there are probably so many children who would benefit from it, and because it’s not a known thing I think a lot of things that children with CVI could be put down to other stuff, like sensory processing stuff, or ADHD. So there’s a lack of awareness amongst professionals, be they teaching or things like speech and language therapy”. Parent 026*

##### Assessment

Parents reported visual needs assessments were often lacking, or not given enough priority in evaluation of support needs.

*“One of my main frustrations is about people not knowing about CVI, never having heard of it, not understanding it. For example, at [school] they have a speech and language therapist who has done a report on [child] and it takes into account sensory stuff about distractibility and stuff, but nothing about CVI, because they don’t know anything about it.” Parent 026*

##### Additional diagnoses

Where additional diagnoses were present, parents felt visual processing was not always given enough weight or attention. Recognition by educational professionals that CVI may occur in conjunction with other conditions was deemed essential so that all aspects of development and learning were addressed in support planning.

*“*…*people hear a word cerebral palsy and they stop looking, and they think that’s fine, they write off his intelligence, I’ve[also] been told that because I have been looking for more diagnosis, I am making him more disabled.” Parent 009*

##### Learning and adaptations

Parents emphasised the significance of recognising the implications of CVI for children’s learning experiences and progress across the whole curriculum (academic, social, and behavioural). Appropriate individualised adaptations were considered crucial for children/young people to thrive, and when present, parents reported positive differences in their child’s learning and emotional wellbeing.

*Researcher: “What sort of things you would say are key?”*

*“The school give her time out, they are fully aware of this CVI*…*.her VI teacher is really keen to make sure that she works, so they have decluttered, everything is very sparse, they put [child] in certain positions that she can see.*…*.she’s got her workspace which is just plain, and with computers and white boards and when she’s in the class they won’t use certain boards because they don’t think it will help her.” Parent 017*

Young people also described the value of appropriate adaptations. This quote from a young person, who is not registered blind, indicates how modifications have helped her.

*“So another thing is that I don’t really read conventionally as in I don’t read like use my eyes to read, I get text to speech on the computer, or I use the headphones to listen to the book.” CYP 006*

##### Training

Parents considered all professionals should be trained to recognise how CVI might affect every aspect of the curriculum; reporting that some teachers had not heard of CVI and were thus unable to provide the support required.

*“Training the schools so they have a better understanding of how to adapt resources and what they can do to help*……*.Yes, if I had a magic wand in an ideal world yes it would, it’s everyone, because the TAs [teaching assistants] are the ones that work one to one with the children, so they need it, but the teachers need it as well so they know what to get the TAs to do.” Parent 028*

##### Specialist support

Visual/sensory support teachers were recognised as vital in supporting teaching staff and providing helpful information.

*“we have really good support from the VI team, they say look this young person needs all this paperwork enlarged, he needs a specific font and specific bold, so the VI teacher was asking [for] the teachers work so she could take it away to get it enlarged*…*.and bring it back so he was actually able to read it.” Parent 025*

Parents viewed their relationships with schools as fundamental to their child’s progress. Input included practical contributions, e.g., making and supplying adapted learning materials or in some cases, reducing or giving up work to create the time and resources to advocate for their child.

*“I feel we were lucky that I took extra time off work with [child’s name], I spent hours, I coordinated getting some consistency and information sharing for [child’s name]. If I hadn’t happened to know how to do that, I don’t think it would have happened.” Parent 012*

Parents described the value of visiting schools to talk to teachers and their child’s class about how CVI might impact their child.

*“I went into his class to do a talk*…*.saying if you want to interact with him you need to come closer, say his name, and then be in front of him, they learnt a lot when I did the talk, because I was able to explain in a suitable format for eight-year-olds that also meant the teacher and the TAs understood a lot more about what was difficult for him.” Parent 004*

Following this, the parent noted:

*“the child’s teacher felt after [parent’s talk] his friends were more tolerant, and one of his friends came and got round to be in front, and I thought it’s obviously clicked with them.” Parent 004*

Young people also described support that helped their learning and other school-based activities including developing skills to support life beyond school.

*“Yeah, because [mobility officer] just helps me go through things, and just being normal and using things people use, and it just helps me break free” CYP 025*.

#### Family Life

Family life discussions focussed on information and support perceived necessary for their child to reach their full potential. In addition to “assessment,” “information,” and “understanding of CVI” described in Theme One, in Theme Three parents emphasised issues more specific for their child to thrive within their family and community.

##### Information

Parents wanted detailed information of how CVI might affect their children’s interactions with their world, not just at diagnosis but at key developmental stages. Information leaflets, videos, or online information, support groups or social media were described as facilitating access to information.

*“Something upfront to say your child has CVI, this is what it means in terms of physical layout, and this is what it means in terms of content of curriculum, here are things that may help, or if there was three guides one for school, one for home and one for out and about, and these are the things that may help you, these are the equipment you may need, or these are the things that you should request, that would have been very useful.” Parent 015*

##### Support

Enabling parents to make necessary adaptations within the home and community was described as essential. Adaptations included physical alterations as well as enablers/carer support to take children out or to play with them at home. Home visits by specialists including IT support; occupational therapists and QTVIs were viewed as beneficial.

*“the community VI teacher organised someone to come to our house and look at his IT stuff*…. *we had some support and help in arranging the desktop icons, the colours and stuff to best suit him as far as we can.” Parent 010*

Physical changes to the home facilitated access and independence. For example, one family moved to a bungalow as their child could not manage stairs. Others reported using location stickers or bright tape to identify door openings or routes through the house; introducing cupboards to reduce/hide visual clutter and putting up “tents” to allow for some “down” time when their children got tired.

*“In terms of CVI, we printed out big red dots, and I asked him where he wanted them stuck round the house, and you do actually see him sometimes tracking to where they are, he uses them to navigate by.” Parent 004*

##### Life at home and the outside world

As part of accessing the outside world, parents described the importance of other people understanding the nature and variability of CVI. For example, for children with additional diagnoses, using a wheelchair for part of the day helped to “preserve” vision for later activities. Support to enjoy local community facilities such as play parks and cinemas was also important, both for the child with CVI and their whole family helping to maintain “normal” family life and activities.

“*sometimes*… *actually we transferred him to sit in his wheelchair for a bit because it preserves his vision for longer in the day, we know that because he’s got the physical and the visual tiring going on, if he tires himself out too much, we know that everything deteriorates*”. *Parent 004*

“*Yeah, every part of that jigsaw needs to fit so that every part of that child’s network understands this, they get that holistic view, and CVI should be a part of it, absolutely should be a part of it.*” *Parent 013*

##### Key people “enablers”

Active involvement and good relationships with professionals including QTVIs, ophthalmologists with knowledge of CVI and occupational therapists were reported as essential and in some cases transformational.

“*I am lucky I wasn’t happy, and I found [QTVI name].*……*via the Internet, Google, just basically I was [looking for] an ophthalmologist for children who can’t communicate, I didn’t know anything about CVI at that point, ‘I need somebody who can deal with a child who has got learning difficulties.’ Thank God I did, because without [name] we would never have a diagnosis.’ Parent 017*‘*We have got the paediatrician she is one of the best ones around in the county, she’s brilliant. I say I want something [clicks fingers] it’s done within*… *brilliant*…*we were struggling with [child’s] walking, but I don’t know whether that is visual as well as a mix with her over supple limbs, but she shuts off, and the paediatrician said, “Would you like a pushchair?” I was like well I don’t know whether it would make a difference, but within two days I had pushed her, and my God it did make a difference, it’s like a comfort blanket for her. So she will walk quite happily, but when something is too much, whether it’s over crowded, she’s in this pushchair and she is a different child, she is back to [child’s name] again.” Parent 017*

##### Supporting independence

Understanding the social world including recognising family and friends; encouraging friendships and negotiating the outside world were all considered important facets of developing independence. These were often facilitated by parents themselves, but additional specific support was also described.

*Researcher: “Is the person from the ‘Guide Dogs’ trying to help with his independence?*

*Oh yes, at the moment, they found that if he wears a baseball cap that helps apparently, helps keep him focused when they are out.” Parent 009*

#### Psychological Wellbeing and Quality of Life

Parents and young people highlighted how their psychological wellbeing and quality of life could be affected by CVI. Parents described the importance of reducing anxiety, increasing social inclusion, reducing frustration, recognising causes of “meltdowns”/tantrums; and increasing self-esteem. Parents acknowledged that some issues their child faced were not solely due to their visual difficulties but involved interactions with other conditions including cerebral palsy or autism.

##### Reduction of anxiety

Parents recounted how their child’s anxieties affected their ability to access many aspects of everyday life, within both community and educational settings

*“when they were doing activities like something crafty, she would find that really stressful because they did everything on the floor, and that didn’t help her at all, she had no defined space. She was worried about being able to find her equipment again if she had to get up and go and get something, that was really stressful for her.” Parent 007*

##### Meltdowns

Parents explained meltdowns often occurred at home when their child had “held everything together at school” or when they just felt overwhelmed coping with the activities of everyday life.

*“I think school do that less well, and that’s why we get the meltdowns, as he gets more tired, like any child would, he’s got extra factors going on.” Parent 004*

*“He said a few things like that, there’s clearly some kind of link between the loud noise and disturbing his vision and things.*…*.he was coming home [from school] and having massive meltdowns, because he is exceptionally good at masking and appearing to be okay and managing, he would hold it together at school absolutely.” Parent 012*

##### Increased social inclusion

Parents expressed the importance of enabling increased participation in social activities at home, in local communities and at school. Successful activities included horse riding, football, swimming, carriage driving, and access to community facilities such as shops and local parks. Support from family and external agencies was described, the latter seen as particularly beneficial for older young people’s independence.

*“he has a personal assistant, and she takes him out and she’s well aware of his needs. So, he has some good quality time with her, she takes him to the cinema, or bowling, or walking or to the park, or to the pub just for a meal.” Parent 025*

One young person also reported how support from a mentor had increased their ability to handle social situations:

*Well basically in the other school, I had a mentor and she helped me to come out of my shell a lot more, and by going through a booklet and saying what to say and what not to say, and basically that gave me that confidence. CYP 025*

##### Reduction in frustration

Reasons for frustration included not being able to join in with everyday activities, challenges for learning and difficulties with social interactions. Reducing frustration was often the goal for strategies to support the child.

*“he was a bright child but couldn’t access the schoolwork, because it was too cluttered, it was written work*,… *it was everything he can’t do, and his behaviour was because he was getting frustrated, and he was getting angry, and they weren’t helping.” Parent 009*

*“[Name] cannot travel independently outside of the home. She is unable to read a bus timetable or plan a bus route or navigate herself to her bus stop. This causes frustration, isolation, limits her ability to be socially included and impacts her self-worth and self-esteem.” Parent 006*

##### Self-Esteem

Parents and older young people described how self-esteem was affected by experiences of managing everyday life with CVI.

*This is another thing that’s coming through all the time, she’s identifying this or her difficulties, as stupidity, and that’s really sad’ Parent 006.*

*“The first thing now is I get accepted as a person, I am not treated like a young child, I am treated like me. But because of the [special] school I’m in even the students they understand me as a normal person*…*and now my self-esteem keeps getting up every time.” CYP 025*

## Discussion

The semi-structured interviews offered parents flexibility to discuss the breadth of their experiences together with describing more detailed aspects of living with CVI. A strength of this study is that young people with CVI were also invited and encouraged to participate where they could, providing valuable insights and perspectives of how their condition affects everyday life.

Parents emphasised the importance of all professionals involved with their child recognising and understanding the complexities of CVI. Linked to this, parents reported that having a diagnosis of CVI within a comprehensive assessment enabled their access to appropriate interventions and support within education and community settings. Some parents reported the difficulties and frustrations of being left to seek out knowledge about their child’s visual difficulties themselves, which were then compounded by professionals (health and education) not accepting this knowledge. Where no diagnosis was offered, or they were offered what they perceived as incorrect or incomplete explanations for their child’s difficulties, parents described how their children/young people were missing out on opportunities that might otherwise be available to them, with the attendant effects on family life.

A recent review of quality of life in parents (whose children had a range of visual impairments) concluded that that parents require better and more extensive information and guidance to understand the diagnosis of their child’s condition, more information about resources and services available and support to manage and adjust to the situation’ (Lupón et al., 2018). McDowell (2020) also notes how crucial timely, accurate and informed diagnosis is to enable parents to become advocates for their child (McDowell, 2020).

Parents’ experiences of their child’s journey through education highlight the gaps they observed in knowledge, assessment and provision of appropriate settings and adaptations. Our study involved a self-selected group of informed parents, who were active in bridging the gaps in provision. However, this leaves open the possibility of a significant inequity for families without the support, resources, and capacity to do the same for their own children. Future research should investigate the potential for inequalities in provision.

Children and young people described their experiences of school, and how appropriate adaptations enhanced or enabled their access to education including the benefits of small teaching hubs, adaptations within the classroom including using tablets and access to appropriate teaching assistant support. Families underlined the importance of all professionals involved with their child understanding the interactions of additional diagnoses with their CVI so that both could be managed effectively.

Accounts of home life including barriers and facilitators to accessing the community and developing independence, along with social inclusion stressed the importance of these aspects of everyday life to parents and young people. Our findings echo other research with young people who have visual impairments and other childhood disabilities, for example the importance for young people of social inclusion and appropriate adaptations to support independence and participation (Foley et al., 2012; Tadić et al., 2015). Our interviews also revealed some strategies that children had developed for themselves to manage their visual environment, including a young girl who changed for PE next to the boys so that she could identify her clothes more easily and a young person who took out most of the “clutter” from her bedroom to moderate her environment.

Children’s and young people’s psychological wellbeing, particularly the importance of understanding and recognising and managing anxiety and frustration was raised across many aspects of everyday life. Parents described the significance of misreading or misunderstanding their child’s behaviour by those around them including teachers, as this could lead to increased distress and “meltdowns” particularly in younger children. For older young people important aspects of psychological wellbeing included not being judged, being accepted as “themselves” and having appropriate support at home and in education to manage challenges faced by CVI and additional diagnoses. Other studies where young people express their concerns and experiences report similar findings including frustration at being restricted in some school activities (Khadka et al., 2012), anxiety about asking for help (Rainey et al., 2016) and lack of understanding from others of visual impairment, especially when it is not an obvious impairment (Tadić et al., 2015). In our study, parents and young people emphasised the importance of self-esteem including the importance of “feeling normal” not being classed as “stupid.”

Many of the families in this study were involved in national support groups or support organisations for families, and children with CVI, and could therefore be characterised as having a greater awareness of the issues raised in the interviews Full demographic information, including socioeconomic status and ethnicity were not collected at the time of interview. For future work it would be important to include a larger sample of families, including inviting those from a wide range of ethnicities and all socioeconomic groups, in order to explore and assess their experiences of CVI in everyday life. The themes reported highlighted many similarities within families’ experiences of everyday life despite the wide geographical area and range of additional diagnoses. Our findings resonate with families’ experiences reported in earlier work (McKillop et al., 2006; Jackel et al., 2010; Jackel, 2019) indicating the continued nature of challenges families still face.

Involving children and young people in exploration of how CVI might affect their everyday lives provided valuable insights into their lived experiences at home and at school. The interviews were designed to include as many children and young people as wanted to participate. Eight children/young people took part in individual interviews. In another four interviews, the children were present some of the time, and joined in with the family interview when encouraged to by their parent/s. The children and young people who took part in individual interviews always had a parent present, and it is possible that this affected their responses. Recent qualitative research that suggests it is important to note that children’s and young people’s concerns are not the same as those reported by their parents and professionals (Rainey et al., 2016). Further work to explore children’s and young people’s experiences of CVI would be valuable.

In conclusion, our interviews demonstrate the value of listening to parents, children, and young people as they describe the wide-ranging impacts of CVI on every aspect of their lives, and how they address the issues that they face daily.

Our interviews provide important first-hand accounts of the impact of living with CVI. Parents described in detail their perspectives of how they enable and support their children to participate in all aspects of their daily life, often bridging the many gaps that they have identified in health care, education, and support.

Our study highlights the need for comprehensive joined up services, where effective communication between families, schools and health professionals, and continuity of support could provide more families with the help they need. A first step toward this would be to increase professionals’ awareness of the possibility of CVI and provide appropriate and timely access for families to comprehensive and multidisciplinary assessments that can identify visual issues.

## Data Availability Statement

The datasets generated for this study are not available, as we do not have consent from families who took part in this study to share these for other research purposes.

## Ethics Statement

The studies involving human participants were reviewed and approved by the University of Bristol Faculty of Health Sciences, Research Ethics Committee (FREC) reference: 58441 December 12, 2017. Written informed consent to participate in this study was provided by the participants’ legal guardian/next of kin.

## Author Contributions

CW devised the study with support from AP and TG. Interviews were carried out by TG, coding and analysis by TG with support from AP. Themes and structure of the final analysis were agreed by all authors. The manuscript was drafted by TG with significant contributions from AP and CW. All authors have agreed this final submitted version.

## Conflict of Interest

The authors declare that the research was conducted in the absence of any commercial or financial relationships that could be construed as a potential conflict of interest.

## Footnotes

1 www.thecviproject.co.uk

2 www.CVIsociety.org.uk

3 http://www.pencru.org/getinvolved/ourfamilyfaculty/

## Supplementary Material

Supplementary Material A Topic guides for parent/carers and young people.

Supplementary Material B Additional illustrative quotes for each theme.
