# Supplementary material for: Bridging the Gap: Parent and Child Perspectives of Living With Cerebral Visual Impairments
Source: Front Hum Neurosci. 2021 Jul 8;15:689683. doi: 10.3389/fnhum.2021.689683 (PMC8295497; doi:10.3389/fnhum.2021.689683)
Supplement: Supplementary Material A — Topic guides for parent/carers and young people. [file Data_Sheet_1.docx]

# Supplementary Material A

**CVI Project Impact Study Qualitative Interviews Topic Guides**

**Topic Guide for Parents or Carers**

**Interview part 1: Day to day life**

**Topics to include:**

**•** Ask about daily routines

• Things they enjoy

• Interests and hobbies

• Social life

• School life

• Family life

• Health and well-being

**Interview part 2: Practicalities**

**Topics to include:**

**•** What’s easy or difficult to manage?

• Things that have changed (since when?)

• Costs – financial, work, etc

**Interview part 3: Support in place**

**Topics to include:**

• What support do they get? From where/whom?

• What works best?

• Day to day support or any future goals (may not be appropriate for some)

**Interview part 4: Support required**

**Topics to include:**

• Struggles or problems faced

• What would have helped?

• Parental stress or worries

• Child / young person stress or worries

• Ideas for help in the future or helping others in similar situation

**Topic Guide for Young People**

**Interview part 1: Daily life**

**Topics to include:**

• Tell me about your day today

• Things they enjoy

• Interests and hobbies

• Social life

• School life

• Family life

**Interview part 2: Support in place**

**Topics to include:**

• What helps you?

• What do you like about the help you get?
