# Supplementary material for: Bridging the Gap: Parent and Child Perspectives of Living With Cerebral Visual Impairments
Source: Front Hum Neurosci. 2021 Jul 8;15:689683. doi: 10.3389/fnhum.2021.689683 (PMC8295497; doi:10.3389/fnhum.2021.689683)
Supplement: Supplementary Material B — Additional illustrative quotes for each theme. [file Data_Sheet_2.docx]

# Supplementary Material B

**CVI Project Impact Study Qualitative Interviews: Additional Illustrative Quotes**

**Themes, sub-themes with additional illustrative quotes from interviews with families.**

| ***Themes*** | ***Illustrative quotes*** |
| --- | --- |
| ***Theme 1: Assessment and understanding of implications of CVI*** | |
| ***Recognition of CVI as a diagnosable condition*** | *‘I’d like for healthcare professionals to recognise it as a condition, and have appropriate clinics, because I think through those clinics you learn from other people. I think that’s how we all learn the strategies,’ Parent 003*  *‘I think my biggest let down is certainly when it comes to the CVI is the fact that [at] the eye tests they said it’s not recognised as a visual problem because it’s brain related…’ Parent 017* |
| ***Professionals’ knowledge*** | *‘I think one of the bigger things we have probably come up against that with other professionals that maybe don’t understand brain issues in neonate and CVI is that we have been told on numerous occasions maybe we should assess for autism a lot.’ Parent 012*  *‘Yeah, it’s great, and increasing her confidence. We are very lucky that we have a consultant who understands CVI so well in our area.’ Parent 005* |
| ***Assessment*** | *‘[hospital professional who carried out] a neuro and psychological assessment, seemed to have such a good grasp of what their difficulties and what their strengths were. having some sort of assessment like that is important because you want to understand what’s going wrong, and I think CVI is quite difficult to unravel and understand what is going on’. Parent 027*  *‘So it was just more to do with the function of his eye and the eye was healthy and all that kind of… more eye health, and a little bit of how he uses his vision and that kind of thing, but it was very much more to check his tracking and his fixing and following and all that kind of stuff. So, from a CVI perspective it was always on his diagnosis list as I say it was never really explained or investigated any further than that really.’ Parent 010* |
| ***Theme 2: Education*** | |
| ***Knowledge*** | *‘I would like to see the VI service incorporate CVI, I think it’s crucial, because there are probably so many children who would benefit from it, and because it’s not a known thing I think a lot of things that children with CVI could be put down to other stuff, like sensory processing stuff, or ADHD. So there’s a lack of awareness amongst professionals, be they teaching or things like speech and language therapy’. Parent 026*  *‘The vision teacher is hoping to get pictures of all the children in the class so that we can go through them at home. They’re going to do the chairs of different colours. They are going to change the coat hook to a brighter colour, she will always have the first hook, and then they are going to paint two of the steps to get out of the school, just a yellow strip.’ Parent 003* |
| ***Assessment*** | *‘even if it’s QTVIs going in and doing assessment and other people delivering it, or schools delivering it, having that actual awareness of the visual issues and knowing… I think my [children] really were probably about 10/11 before they had any decent assessment, and by that point your education is gone, you have missed the window.’ Parent 011*  *‘Or the link person from RNIB, and I know she was at the clinic where they did the diagnosis of the CVI, that if she essentially is the linchpin so that if there was something similar in terms of education as well, so whereas that clinic was brilliant and that it brought neurology, OT, and all orthotics and everything else together, it would have been good to have someone from education there as well.’’ Parent 015* |
| ***Additional diagnoses*** | *‘he just got picked up because he wasn’t doing any of the major milestones, he wasn’t rolling over, he wasn’t crawling, so we got picked up by physiotherapy earlier and he had a walking frame, and he was walking when he was three, At that point we got engaged with neurology and had some scans and things, but we’ve had so many tests, blood tests, genetic tests, we haven’t really got anything, we’re still one of the 100,000 genome tests that going on, but nothing.’ Parent 029.*  *‘there was a host of other things going on, I am sure a lot of children do and that’s why CVI probably gets missed, because he has balance and coordination difficulties with no diagnosis for these, just having things that him. So in fact it wasn’t until he was five he went to school that we knew he had vision problem at all.’ Parent 029* |
| ***Learning and adaptations*** | *‘The problem we have is …the level of work that’s taking place within the classroom is far too advanced, and the sound and the visual clutter in the room, they’ve tried to cut down the visual clutter a little bit, but what seems to happen is every year you move to another classroom and it all goes back to exactly how it was before and you have the same battle.’ Parent 005*  *‘She usually helps me do stuff like if I’m stuck with something she will always help me, she basically reads the questions out because if I am struggling because I can’t always see, even though I’ve got my glasses on I still can’t even see the small words, so she has to read them out to me.’ Child/Young Person 011* |
| ***Training*** | *‘I think the thing is that they needed the training and the understanding, and it needed to be a specialist resource and trained staff.’ Parent 011*  *‘they [CVI Society] came and did an awareness raising session in the school, which was incredible and did a presentation, So there was a better understanding in the school and I think this is something that needs to be done to the whole school and all the staff who work with that child.’ Parent 005* |
| ***Specialist support*** | *‘they have just signed up for or they are navigating RNIB book share, and they’re in the beginning stages of getting to grips with that. She has a reading assistant that she been working with once a week, one to one, and will be working with three times a week next year, she’s going to really be the person that monitors whether it’s impacting, and how it’s impacting on her reading as she is dealing with more complex texts.’ Parent 007*  *in their view her acuity is corrected by glasses, so therefore she is not eligible for any support from the sensory services, which means, we’ve got support in place for her, but it’s not the right support. It enables one to one support all the time…however, she’s a visually impaired child who has no visual support, and I think we’ve got a real problem here because children with CVI aren’t recognised as having a vision disorder.’ Parent 005* |
| ***Theme 3: Family Life*** | |
| ***Information*** | *‘Somebody to provide information, somebody to come to your house and just talk through the things that a child with CVI might find difficult, because I have worked it out on my own and through reading and stuff, but it would be really nice if I could have a person doing that.’ Parent 007*  *‘A lot of young people do have their own voice if they are able, and it’s about specifically professionals….paediatricians and ophthalmologists or other doctors making any information that’s given to our young people, very plain English. via braille, or read it via large print’ Parent 025*  *‘To try to separate out what’s living with CVI is impossible to say because it’s just in the mix with everything else that’s going on, and actually what I would love is just someone to sit down who is an expert, all the things they had and say okay this is what’s really going on, because you get neurology appointment, you go to your ophthalmology appointment, and they all talk about their little bit, so nobody really helps you understand the whole picture.’ Parent 029* |
| ***Support*** | *‘If there’s a VI charity in the area that’s operating, making sure that people are getting referred to it and that somebody is making contact with them, and they’ve got somewhere to go when they get stuck, I think the third sector support has got a massive role for parents, and I think with visual impairment it’s underutilised. It took me four years to find out that I could join the RNIB, and the RNIB has been amazing, the RNIB has been perfect, but I didn’t know we could join, nobody told me you could join if you’ve got CVI.’ Parent 011*  *I think when he first had his diagnosis, we would have liked more support. We would have liked to have known or be introduced to various charities……well we didn’t even know about ‘Sense’ for goodness sake, so we have never joined, Parent 023* |
| ***Life at home and the outside world*** | *‘But I think the main things are things like we always take the iPad so we can photograph things in a distance for him to see. So on a family day out he can’t see the snow leopard against the white background, but when we photograph it and enlarge it we can say and there’s the snow leopard, it’s over there.’ Parent 004*    *‘We ourselves would have had sight tape everywhere because she would have been bumping into doors and skirting boards and lots of things, so we would have had the whole ground floor of the house taped at her sight level anyway, and we would have been using things like differentiation in colour between if we were placing a bowl on top of a placemat.’ P015*  *‘Interestingly one day, she had lots of posters and things all over her walls, stickers, or flowers and things, and then one day, a year ago, initially she had [taken] down everything on her walls. She couldn’t explain to me why, and then she wanted some things put back up, but it’s a lot less. So it’s almost like she regulated her own environment.’’ Parent 026* |
| ***Key people ‘enablers’*** | *‘How a child without other visual issues would ever get diagnosed with CVI I can’t see it happening, because without the QTVI coming to see us anyway [for nystagmus], it was her expertise when she said ‘I think we might be looking at[CVI]… she wouldn’t have been anywhere near our family if it wasn’t for the nystagmus.’ Parent 012*  *‘We’re very fortunate in that we have an OT who has direct experience of CVI, so this OT is able to say she can’t have a desk next to the window it’s too distracting, she can’t be next to the door, she needs to sit here.’ Parent 005* |
| **Supporting independence** | *‘That gave us a Blue Badge and stuff like that, also they gave us some support around the home, talking jugs and talking bowls, , and support to guide him in the community, that was really important, and upskilling [name] to be able to go out in the community as well.’ Parent 025*  *‘I also using the RNIB forum a lot because it gives me help to understand and how to understand that, because RNIB is helpful because you can buy stuff off there for blind people, so liquid level indicators because when you pour drinks and you can’t see the water, you can just use the thing and beep, beep, beep, when it’s time to stop.’ CYP 006* |
| **Theme 4: Psychological wellbeing and quality of life** | |
| **Reduction of anxiety** | *‘I think in general she has strategies to manage pretty much everything. One of the things she told me she does at school which nobody had realised was that she deliberately gets changed for PE near the boys so that her clothes don’t get muddled up because the boys’ clothes are different, so her school dress is completely different’. Parent 007*  *‘I think routine is key as well, so as long as she knows where she’s going, what she’s doing, who is going to be there, and she will ask you these questions a million times a day, , because she does get anxious, and then what she is doing it afterwards, that nails it to start off with as long as she’s got that in her head.’ Parent 017* |
| **Meltdowns** | *‘She has CVI meltdowns where she will just get very overwhelmed and upset, and crying, and leave me alone, and shouting’. Parent 005*  *‘With colours, which was why the colour blindness, he had a meltdown the other day, apparently I was using the base of his toothbrush, now mine is pink and aqua and his is blue and red, primary colours, because he saw the pink as red, from where he was looking, and it took me to take it out of the light and put it by him to show him and he still said, “That looks red to me.” And yet other times he will see it as pink.’ Parent 009* |
| **Increased social inclusion** | *‘Yeah, so friends withdraw as well because they are like well, I don’t want to invite you to the party because [children] won’t cope with it, because it’s a laser quest or in the dark, or we’re going to the cinema, and so it does have a massive effect on relationships, you end up quite isolated I think.’ Parent 027*  *‘But as an independent young person he doesn’t want to explain himself ten times a day, if he’s got a card that he can produce out of his wallet to say you have a little read of this and this will explain what support I need, and he will tell them what support he needs.’ Parent 025*  *‘On Sundays in the morning I go to football,. it’s good. And the good thing about it as well it’s not competitive, there’s just a bunch of people…and that’s why I like it, it’s not competitive, it’s for fun, it’s totally for fun.’ CYP 027* |
| **Reduction in frustration** | *‘Actually, that does frustrate him, his younger brothers can do everything quicker than him, faster than him, and there’s quite a lot of stress because all he has got really is physical force, he’s bigger than them, he’s older than them, but they’re quicker and not particularly sympathetic to him. They don’t give him time to process things that they know it takes him longer, but we try and… they are not mean to him, but they don’t cut any slack.’ Parent 029*  *‘Trying to get them to recognise that what they were giving him was sensory overload, and then as he progressed up the school that he was a bright child but couldn’t access the schoolwork, because it was too cluttered, it was written work, it wasn’t… it was everything he can’t do, and his behaviour was because he was getting frustrated, and he was getting angry, and they weren’t helping. Parent 009* |
| **Self esteem** | *‘Yeah, because [mobility officer] just helps me go through things, and just being normal and using things people use, and it just helps me break free, maybe completely break free from disability, but it does help me get over a few things.’ CYP 025*  *‘[name] life would have been very different. We found that when he was five and up until that point he was completely nothing, we’d not found anything worked, nothing, and his life was awful, and he was awful, and he was so unhappy, and then he found something that he could do, and he was really… he is good, and I think that just it changed everything for us, because we learned to believe that he could do it. And then because of that we were able to make successes in other places’. Parent 011* |
